# Survival Patterns of Patients with Ovarian Cancer in Africa: Systematic Review and Meta-analysis

**DOI:** 10.1245/s10434-026-19413-7

**Published:** 2026-03-18

**Authors:** Chalie Mulugeta, Tadele Emagneneh, Nigusie Abebaw, Aynalem Yetwale, Tilahun Wodaynew, Abebaw Alamrew

**Affiliations:** 1 Department of Midwifery, College of Health Science, Woldia University, Woldia, Ethiopia; 2 Department of Nursing, College of Health Science, Woldia University, Woldia, Ethiopia; 3 Department of Midwifery, College of Medicine and Health Science, Wollo University, Dessie, Ethiopia

**Keywords:** Ovarian cancer, Survival analysis, Systematic review, Meta-analysis, Africa

## Abstract

**Background:**

Ovarian cancer is among the most fatal gynecologic malignancies worldwide, with marked regional disparities in survival outcomes. In Africa, limited comprehensive data exist on survival rates, yet available evidence highlights late-stage diagnosis, restricted access to specialized oncology services, and inequities in treatment availability as key contributors to poor prognosis. Understanding these survival trends is critical not only for clinical awareness, but also for informing health system planning and guiding policy decisions aimed at improving cancer care infrastructure, resource allocation, and patient outcomes across the continent. This meta-analysis synthesizes current data to estimate 1-, 2-, 3-, 5-, and 7-year survival rates for patients with ovarian cancer in Africa, providing an essential benchmark for policymakers and healthcare providers to address gaps and disparities compared with global survival patterns.

**Materials and Methods:**

A systematic review of cohort studies was conducted from 1 to 29 February 2025, by using PubMed, Hinari, EMBASE, Google Scholar, and Web of Science. Methodological quality was assessed using the Newcastle–Ottawa 2016 Critical Appraisal Checklist. Publication bias was evaluated using funnel plots and Egger’s test, and heterogeneity was assessed with the *I*-squared test. Data were analyzed with Stata 11.

**Results:**

A total of 38 articles, including 7339 participants from African countries, were included in this review. Using a random-effects model, the pooled survival rates were estimated as follows: the 1-year survival rate was 71.41% (95% CI 63.71–79.11%); the 2-year survival rate was 69.08% (95% CI 57.31–80.85%); the 3-year survival rate was 61.49% (95% CI 45.19–77.80%); the 5-year survival rate was 61.73% (95% CI 52.72–70.74%); and the 7-year survival rate was 53.74% (95% CI 34.24–73.25%).

**Conclusions:**

This meta-analysis provides a broad overview of ovarian cancer survival rates in Africa, showing variation across timepoints and geographic regions. While the estimated survival rates are encouraging, they remain lower than those reported globally, highlighting ongoing challenges related to late diagnosis, limited treatment access, and healthcare disparities. Policymakers should prioritize raising awareness of cancer symptoms, enhancing early diagnosis through effective referral systems, investing in workforce capacity for specialized surgery and oncology care, and ensuring equitable access to quality cancer services. Further research and context-specific policy measures are essential to support sustainable improvements and the standardized International Federation of Gynaecology and Obstetrics (FIGO) staging system and World Health Organization (WHO) guidelines protocol in survival outcomes across the continent.

**Supplementary Information:**

The online version contains supplementary material available at 10.1245/s10434-026-19413-7.

Ovarian cancer is one of the most fatal gynecologic malignancies worldwide, ranking as the eighth most commonly diagnosed cancer and the fifth leading cause of cancer-related deaths among women.^1^ According to GLOBOCAN 2022, there were approximately 324,603 new cases and 206,956 deaths worldwide, with projections estimating a more than 55% increase in incidence and nearly 70% increase in deaths by 2050.^2^ Unlike cervical cancer, ovarian cancer lacks effective screening programs, resulting in late-stage diagnosis in about 75% of cases, which contributes to the poor global 5-year survival rate ranging from 30 to 50%, depending on healthcare access.^3–5^ In contrast, the USA reports a 5-year relative survival rate of up to 80% for specific stages due to early detection and advanced treatments.^6^

Almost all ovarian tumors originate from one of three types: epithelial cells (90%), stromal cells (5–6%), and germ cells (2–3%).^7^ Established risk factors include older age, genetic predisposition, infertility treatments, and family history, whereas pregnancy, lactation, and oral contraceptive use offer some protection.^8^ By eliminating modifiable risk factors such as obesity, smoking, and the use of hormone replacement therapy, the incidence of ovarian cancer could potentially be reduced by one-third to two-fifths.^9^

Treatment advances, including cytoreductive surgery, platinum-based chemotherapy, and targeted therapies such as poly (ADP-ribose) polymerases (PARP) inhibitors have improved survival in high-income countries.^10^ The 5-year survival rates vary widely: 36–46% in high-income countries^11^ versus below 30% in most low- and middle-income countries (LMICs) due to late-stage diagnosis, limited access to specialized oncology care, and financial barriers.^12–14^ More than 75% of ovarian cancer cases in LMICs present at an advanced stage, compared with 50% or lower in developed nations.^15^ Projections indicate that by 2040, new cases and deaths will increase by 96% and 100%, respectively, in low Human Development Index (HDI) countries, compared with 19% and 28% in very high HDI countries.^16^

In Africa, ovarian cancer incidence is increasing, yet survival rates remain alarmingly low, largely due to delayed presentation, health seeking behavior, constrained healthcare resources, and the absence of standardized treatment protocols.^17,18^ Key challenges to improving survival in African settings include late stage diagnosis,^14,19,20^ limited access to treatment,^21^ economic and healthcare disparities,^19^ suboptimal cytoreduction,^22^ adjuvant chemotherapy,^23^ lymph node metastases,^14^ older age,^20^ and presence of ascites.^20^

Understanding ovarian cancer survival in Africa is limited due to fragmented data, late diagnosis, and inadequate treatment infrastructure. This systematic review and meta-analysis synthesizes existing evidence across African regions to identify survival patterns and highlight gaps in care. The findings aim to inform policymakers, support national cancer control strategies, and improve oncology resource allocation.

Therefore, this study aimed to estimate the survival pattern of 1-, 2-, 3-, 5-, and 7-year survival rates of ovarian cancer in African countries and explore variations across countries, over time, and by development status.

## Methods and Materials

### Study Protocol and Reporting

This systematic review and meta-analysis focused on cohort studies examining the survival pattern of patients with ovarian cancer in Africa. This systematic review and meta-analysis were carried out per the Preferred Reporting Items for Systematic Reviews and Meta-Analyses (PRISMA) criteria^24^ (Supplementary S1 file). The review protocol was retrospectively submitted to International Prospective Register of Systematic Reviews (PROSPERO). The eligibility criteria were adapted from the Newcastle–Ottawa 2016 review guidelines.^25^

### Inclusion Criteria

We searched for eligible human studies published in English. All quantitative studies that reported variables or indicators related to the survival rate of patients with ovarian cancer were included in the systematic review and meta-analysis. The review considered all cohort studies written in English and conducted in Africa. The literature search for this systematic review and meta-analysis was conducted between 1 and 29 February 2025; however, no restriction was applied to the publication date of the included studies. The primary outcome measure was 1-, 2-, 3-, 5-, and 7-year survival rates.

### Exclusion Criteria and Missing Data Management

Studies were excluded if they were anonymous reports, defined as documents with no identifiable author, institutional affiliation, or publication source. These included grey literature and online documents without peer review or verifiable origin, which could not be assessed for methodological quality or credibility. Additionally, we excluded studies conducted outside of Africa, reviews, case reports, editorials, and studies lacking relevant survival data. Studies focusing solely on specific factors or descriptive frequency analyses were also excluded, as they did not provide concrete survival data necessary for this review.

For included studies, if a study did not report survival outcomes for a specific timepoint (1-, 2-, 3-, 5-, or 7-year survival), the data from that study were not included in the meta-analysis for that timepoint. No imputation of missing outcomes was performed. All other available data were included, and sensitivity analyses were conducted to ensure that the exclusion of studies with missing outcomes did not substantially alter the pooled survival estimates.

### Operational Definition

Ovarian Cancer was defined as a malignant tumor that originates in the ovarian tissue, often classified into epithelial, germ cell, and stromal tumors.^26^ Survival rate refers to the probability of patients remaining alive for a specified period following diagnosis. It is estimated using time-to-event data through survival analysis techniques, commonly the Kaplan–Meier method, which calculates survival time from the date of diagnosis to the date of death or last contact for those who are censored (still alive or lost to follow-up at the time of analysis).^27,28^ Advanced stage ovarian cancer was disease classified as stage III or IV on the basis of the International Federation of Gynaecology and Obstetrics (FIGO) staging system, characterized by extensive spread beyond the ovaries.^29^

### Search Strategy

A comprehensive and reproducible search strategy was developed and implemented in accordance with the PRISMA 2020 guidelines. We systematically searched PubMed, EMBASE, Web of Science, Hinari, and Google Scholar to identify relevant studies reporting survival outcomes among patients with ovarian cancer in Africa. An initial exploratory search was conducted to identify relevant keywords and Medical Subject Headings (MeSH). On this basis, a final structured search strategy was developed. The PubMed search strategy included the following terms: (“Ovarian Neoplasms” [MeSH] OR “ovarian cancer” OR “ovarian carcinoma”) AND (“Survival” OR “Survival Rate” OR “Overall Survival” OR “survival analysis”) AND (“Africa” OR “Sub-Saharan Africa”).

Boolean operators (AND, OR) were used to combine search terms, and equivalent terms were adapted for each database. No restriction on publication year was applied. Only studies published in English were included. The final search was conducted on 29 February 2025. Full database-specific search strategies are provided in Supplementary File S2.

### Data Extraction

Data extraction^30^ was conducted independently by two reviewers. AA and TE conducted the initial screening by assessing the titles and abstracts of identified studies. Full-text screening was performed for potentially eligible articles. A standardized data extraction form was used to capture study characteristics, including author, publication year, country, study design (prospective or retrospective), follow-up duration, and sample size. From each included study, we extracted the survival rates at available timepoints (1-year, 2-year, 3-year, 5-year, or 7-year survival) for a single cohort of patients with ovarian cancer.

Survival outcomes included overall survival (OS) rates at 1, 2, 3, 5, and 7 years, as reported in the individual studies. Where available, point estimates and 95% confidence intervals (CIs) were extracted. Any disagreements were resolved through discussion and consensus. If consensus was not achieved, a third reviewer adjudicated the disagreement.

### Quality Assessment/Critical Appraisal

The inclusion and exclusion criteria were applied to review the remaining articles, focusing on the pattern of survival rate among patients with ovarian cancer in Africa. The Newcastle–Ottawa quality appraisal checklist was used to evaluate the quality of individual studies^25^ (Supplementary S3 file Table 1). The quality of each included study was evaluated using the Newcastle–Ottawa Scale (NOS) for cohort studies. The NOS assesses studies on three main domains: selection of participants, comparability of study groups, and assessment of outcome. To ensure the reliability and validity of included studies, the Newcastle–Ottawa Scale (NOS) was used for quality assessment. The NOS evaluates observational studies on the basis of three domains: the selection of study participants (four points), the comparability of study groups (two points), and the outcome assessment (three points). Each study was rated on a scale of 0 to 9, with studies scoring ≥ 6 considered high quality and those scoring < 6 deemed low quality. Quality assessment was conducted independently by two reviewers (AA and TE), with discrepancies resolved by consensus. Each cohort study was assessed using eight criteria: inclusion criteria, study subject and setting description, valid measurement of exposure, and identification of confounders using objective criteria, confounder handling strategies, outcome measurement, and statistical analysis. A total of 38 cohort studies met the quality criteria and were included in the analysis. To incorporate risk of bias into our synthesis, we considered NOS scores in both study inclusion and interpretation. Studies with NOS scores < 6 were regarded as having a higher risk of bias and were excluded from the meta-analysis. Furthermore, we conducted sensitivity analyses to assess the impact of study quality on pooled estimates, and findings were interpreted with consideration of study-level quality and potential sources of bias.

### Data Synthesis and Statistical Analysis

The extracted data were synthesized quantitatively through meta-analysis where feasible. Pooled survival estimates at 1, 2, 3, 5, and 7 years were calculated using reported survival rates and their corresponding 95% confidence intervals (CIs). A random-effects model (DerSimonian–Laird method) was employed when substantial heterogeneity was detected (*I*^2^ > 50%).

Heterogeneity among included studies was assessed using the *I*^2^ statistic, with values categorized as low (*I*^2^ < 25%), moderate (25–50%), or high (> 50%). Sensitivity analyses were performed to evaluate the robustness of the findings by excluding studies with a high risk of bias or influential outliers. Subgroup analyses were conducted on the basis of geographical region and period of publication to explore potential sources of survival variations. Studies were grouped into four African regions: Northern, Western, Eastern, and Central Africa. One study (Gizaw et al.) reported data exclusively for Sub-Saharan Africa without further regional disaggregation; to maintain data integrity and contextual accuracy, Sub-Saharan Africa was retained as a separate category in the regional subgroup analysis. Publication bias was assessed through funnel plots and Egger’s test, and whether significant bias was detected.^31^

The certainty of evidence for each survival outcome was assessed using the Grading of Recommendations, Assessment, Development and Evaluations (GRADE) approach. The following domains were evaluated: risk of bias, inconsistency, indirectness, imprecision, and publication bias. Risk of bias was assessed using the Newcastle–Ottawa Scale for observational studies. Observational studies start as low certainty by default, and certainty could be downgraded for serious limitations or upgraded for factors such as large effect size or narrow confidence intervals. Heterogeneity was quantified using *I*^2^ statistics, and imprecision was evaluated on the basis of the width of 95% confidence intervals and the number of included studies.

### Software

We used EndNote (version X7) reference management software to download, organize, and review; Zotero to cite related articles;^32^ and STATA version 11^33^ for statistical analyses. These software tools were used for meta-analysis, generating forest plots, and performing statistical tests for heterogeneity and publication bias.

### Reporting

The review’s findings were reported following PRISMA guidelines. Results were presented in both narrative and tabular formats, with meta-analysis outcomes depicted in forest plots for 1-, 2-, 3-, 5-, and 7-year survival rates.

## Results

### Searching Findings

**Fig. 1:**
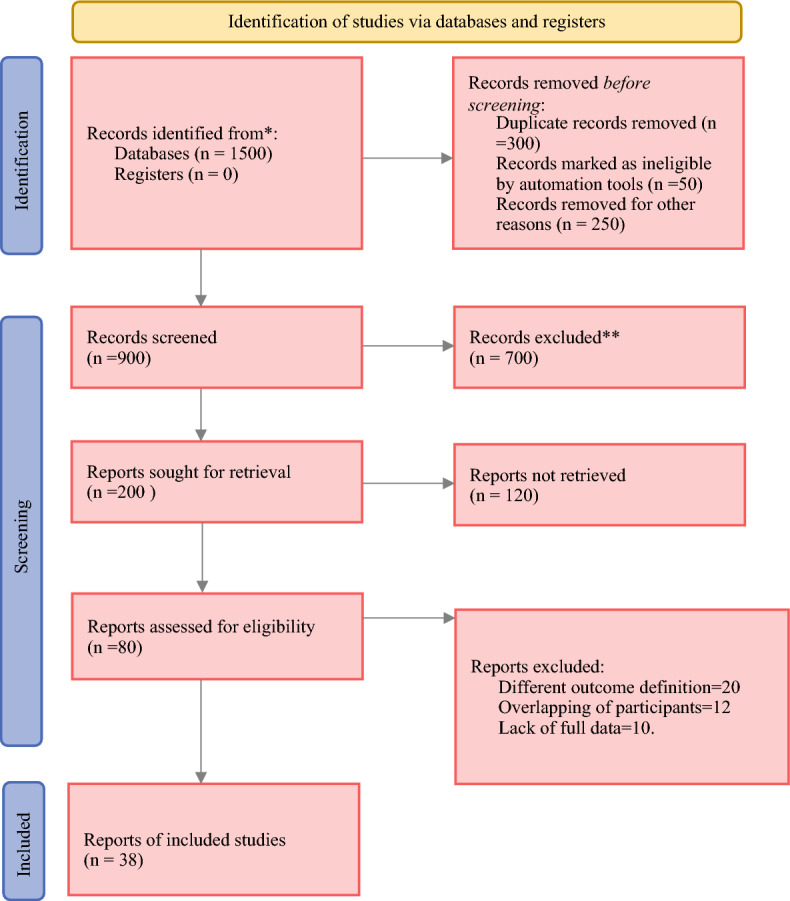
PRISMA flowchart diagram of the study selection process

A total of 1500 published studies (PubMed 500, Hinari 20, Google 100, EMBASE 10, Google Scholar 800, Web of Science 70) were identified. A total of 600 records were removed, comprising 300 duplicate records, 50 records flagged as ineligible by automation tools, and 250 records removed for other reasons, leaving 900 abstracts for evaluation. A total of 700 articles were excluded on the basis of methodological criteria issues, not focused on Africa, and not relevant to ovarian cancer. A total of 200 articles were sought for retrieval, resulting in 120 articles that were not retrieved. Finally, 80 articles were eligible for full-text screening, and 42 articles were further excluded for various reasons, leaving only 38 studies for final systematic review and meta-analysis (Fig. 1). The subset of studies included in this report was published between 2012 and 2025.

### Study Characteristics

A total of 38 studies were included in this analysis. Of the 38 articles included in the systematic review, 18 articles were conducted in Egypt,^34–51^ 7 in Nigeria,^52–58^ 1 article in Botswana,^59^ 2 articles in Ethiopia,^60,61^ 1 article in Tanzania,^62^ 1 article in Sudan,^63^ 1 from 6 countries’ cancer registers,^64^ 6 in Kenya,^65–70^ and 1 in Algeria.^71^ The subset of studies included in this review was published between 2012 and 2025, and 85% of the included articles were published after 2015. From the included articles, 7339 patients with ovarian cancer participated in identifying the survival rate. The included articles were published. All the included studies were facility-based cohort studies by design and reported survival rates of patients with ovarian cancer. The sample sizes across the studies ranged from 22^47^ to 644^64^ (Table 1). The average age of patients with ovarian cancer was 47.37 years.

**Table 1:** Characteristics of included studies with 1-, 2-, 3-, 5-, and 7-year survival rates of patients with ovarian cancer in Africa: systematic review and meta-analysis

Author	Country	Actual sample	Period of publication	Average age	1-year survival rates	2-year survival rates	3-year survival rates	5-year survival rates	7-year survival rates
Mazouzi C, et al.	Algeria	20	2023	50			38.71%		
Lumley C, et al.	Botswana	38	2023	57	62.8 %	43.7 %			
Elashry R, et al.	Egypt	42	2013	11.26				76.2 %	
Sallam YA, et al.	Egypt	265	2016	48				85 %	
Nabil H, et al.	Egypt	94	2020	50	80.9 %				
Zuhdy M, et al.	Egypt	42	2023	50.17				78.5 %	
Nassar HR, et al.	Egypt	174	2015	50		94.8 %		51.3 %	
Bassiouny D, et al.	Egypt	166	2018					70 %	
Fayek IS, et al.	Egypt	77	2019	47.34		97 %	80 %	50 %	45 %
Ali A, et al.	Egypt	40	2017	9.5					77.5 %
Elzarkaa AA, et al.	Egypt	96	2018	57 %		39.6 %			
Kamal IM, et al.	Egypt	85	2020	53 %					22.2 %
Amin NH, et al.	Egypt	80	2023						63.8 %
Gohar S, et al.	Egypt	66	2019	52 %			97 %		
Abdelrahman M, et al.	Egypt	38	2023	55 %	75 %				
Saber MM, et al.	Egypt	19	2014	23 %				93.8 %	
Sheta H, et al.	Egypt	98	2021	57.1 %			33 %	58.3 %	
Mostafa MF, et al.	Egypt	116	2012	47 %		72 %			
Abdel Ghany AE, et al.	Egypt	83	2014	55 %					61.4 %
Piszczan S, et al.	Ethiopia	485	2019	46 %	75 %	59 %			
Konya WP, et al.	Kenya	55	2021	46 %		73 %		55.6 %	
Mayenga DB, et al.	Kenya	112	2024	51.28 %				44 %	
Cheserem EJ, et al.	Kenya	224	2013	49 %		50 %			
Mburu AW, et al.	Kenya	29	2022			65 %			
Mworia KM, et al.	Kenya	44	2023	49.3 %		91 %		81 %	
Mokomba A, et al.	Kenya	180	2021	50 %		74 %		45 %	
Ayogu ME, et al.	Nigeria	65	2020	50.2 %				84.5 %	
Okunade KS, et al.	Nigeria	50	2016	45.7 %				64 %	
Okunade KS, et al.	Nigeria	134	2024	51.5 %			59.5 %		
Okunade KS, et al.	Nigeria	83	2025	54.4 %		65.1 %			
Okunade KS, et al.	Nigeria	176	2022	50.6%			63.6 %	43.3 %	
Okunade KS, et al.	Nigeria	93	2022	47.1%			61.3 %		
Iyoke CA, et al.	Nigeria	54	2013	45.4%	30%				
Abuidris DO, et al.	Sudan	341	2016	50 %				38%	
Mlagalila NF, et al.	Tanzania	96	2017	51.4%	82%				
Gizaw M, et al.	Sub-Saharan (6Countries)	644	2024	48.2%	73.4%		54.4%	45%	
Hegazi R, et al.	Egypt	95	2013	52.2%	83%				
Habteyes AT, et al.	Ethiopia	561	2025				38.71%	50.22%	

### Meta-analysis

#### Pooled 1-Year Survival Rate

**Fig. 2:**
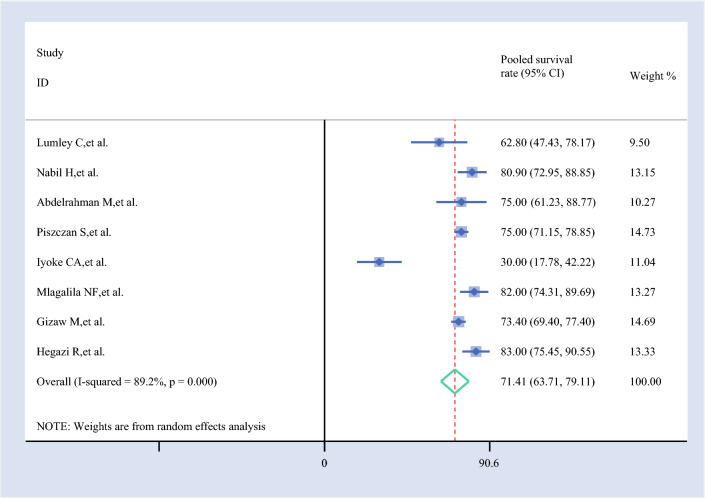
Forest plot of the 1-year pooled survival rate of patients with ovarian cancer in Africa

The 1-year survival rate for ovarian cancer, based on eight studies, was 71.41% (95% CI 63.71–79.11%). This indicates a moderate survival outcome for patients with ovarian cancer within the first year after diagnosis, as derived from the pooled data of the included studies (Fig. 2).

#### Pooled 2-Year Survival Rate

**Fig. 3:**
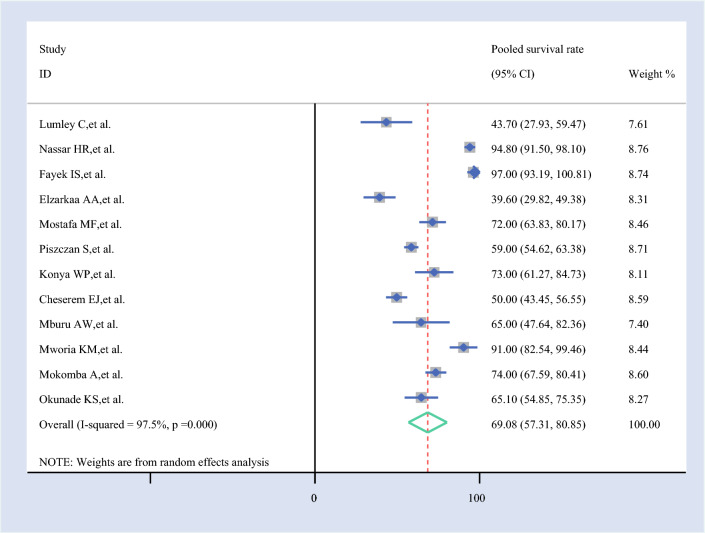
Forest plot of the 2-year pooled survival rate among patients with ovarian cancer in Africa

The 2-year survival rate for ovarian cancer, based on 12 studies, was 69.08% (95% CI 57.31–80.85%). This reflects the overall survival outcome for patients with ovarian cancer after 2 years, as estimated from the pooled data of the included studies (Fig. 3).

#### Pooled 3-Year Survival Rate

**Fig. 4:**
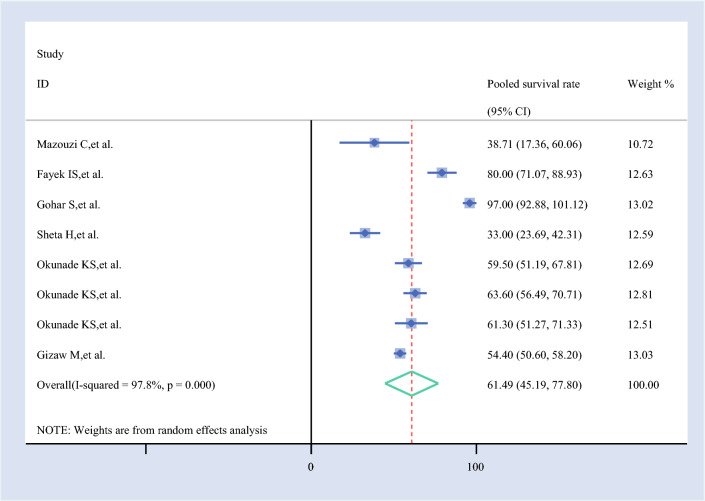
Forest plot of the pooled 3-year survival rate among patients with ovarian cancer in Africa

The 3-year survival rate for ovarian cancer, based on eight studies, was 61.49% (95% CI 45.19–77.80%). This indicates a moderate survival outcome for patients with ovarian cancer at the 3-year mark, as derived from the pooled data of the included studies (Fig. 4).

#### Pooled 5-Year Survival Rate

**Fig. 5:**
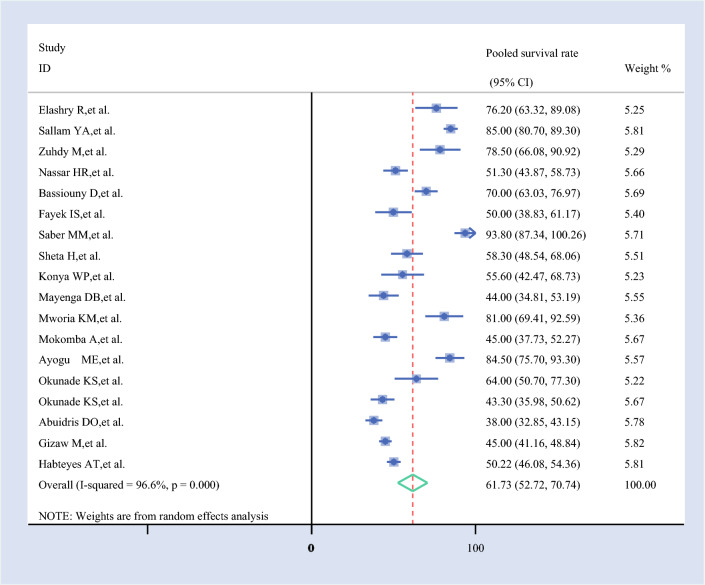
Forest plot of the pooled 5-year survival rate among patients with ovarian cancer in Africa

The 5-year survival rate for ovarian cancer, based on 18 studies, was 61.73% (95% CI 52.72–70.74%). This suggests a moderate survival outcome for patients with ovarian cancer at the 5-year mark, as estimated from the pooled data of the included studies (Fig. 5).

#### Pooled 7-Year Survival Rate

**Fig. 6:**
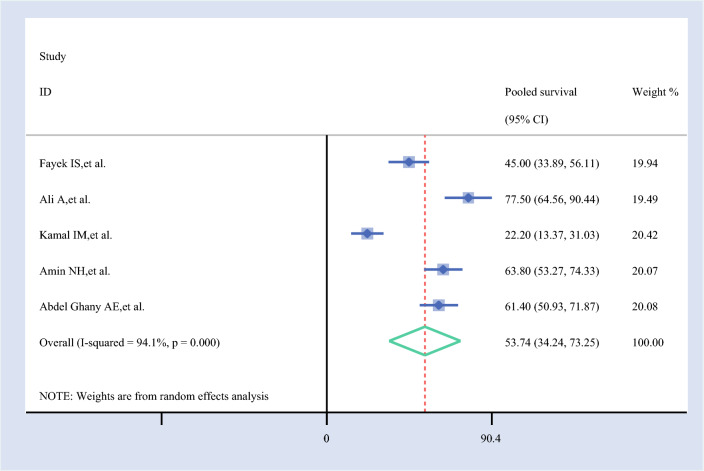
Forest plot of the pooled 7-year survival rate among patients with ovarian cancer in Africa

The 7-year survival rate for ovarian cancer, based on five studies, was 53.74% (95% CI 34.24–73.25%). This indicates a relatively lower survival outcome for patients with ovarian cancer at the 7-year mark, as derived from the pooled data of the included studies (Fig. 6).

### GRADE Certainty

The certainty of evidence for the pooled survival rates of patients with ovarian cancer in Africa was rated as moderate for the 1-, 2-, 3-, and 5-year outcomes and low for the 7-year outcome (Table 2). These ratings were determined using the GRADE approach, which evaluates risk of bias, inconsistency, indirectness, imprecision, and publication bias.

**Table 2:** Pooled survival rates of patients with ovarian cancer in Africa and certainty of evidence according to GRADE

Outcome (survival rate)	No. of studies	Participants	Pooled survival rate (95% CI)	Certainty of evidence (GRADE)	Comments
1-year	8	1191	71.41% (63.71–79.11%)	Moderate	Very high heterogeneity (*I*^2^ = 89.2%), narrow CI; observational studies
2-year	12	1605	69.08% (57.31–80.85%)	Moderate	Very high heterogeneity (*I*^2^ = 97.5%), narrow CI; observational studies
3-year	8	1038	61.49% (45.19–77.80%)	Moderate	Very high heterogeneity (*I*^2^ = 97.8%), narrow CI; observational studies
5-year	18	3422	61.73% (52.72–70.74%)	Moderate	Very high heterogeneity (*I*^2^ = 96.6%), narrow CI; observational studies
7-year	5	383	53.74% (34.24–73.25%)	Low	Very high heterogeneity (*I*^2^ = 94.1%), wide CI

Although the included studies were of moderate-to-high quality according to the Newcastle–Ottawa Scale, all were observational in design, which in GRADE automatically starts as low certainty. For the 1- to 5-year survival outcomes, the relatively narrow confidence intervals allowed a partial upgrade to moderate certainty, despite very high heterogeneity (*I*^2^ = 89–97%). High heterogeneity reflects substantial variability in patient populations, treatment access, and healthcare infrastructure across different African countries, and this inconsistency prevents a rating of high certainty. The 7-year survival outcome was rated as low certainty, primarily due to wide confidence intervals and few contributing studies (*n* = 5). The combination of very high heterogeneity (*I*^2^ = 94.1%) and imprecision limits confidence in this long-term survival estimate.

### Heterogeneity

Heterogeneity in this meta-analysis was assessed using *I*^2^, which quantifies the proportion of variation in study results due to true differences rather than chance. High *I*^2^ values indicate substantial heterogeneity, reflecting variability in survival estimates across studies arising from differences in population characteristics, tumor types, disease stages, healthcare infrastructure, and treatment protocols. The observed heterogeneity was statistically significant (*p* < 0.001) across all survival timepoints. Specifically, 1-year survival exhibited an *I*^2^ of 89.2%, 2-year survival 97.5%, 3-year survival 97.8%, 5-year survival 96.6%, and 7-year survival 94.1%, indicating considerable variability among studies. This high heterogeneity underscores that the pooled survival estimates represent a common measure across heterogeneous patient populations, and caution should be exercised when interpreting these results for specific subgroups. Differences in geography, access to care, diagnostic capacity, tumor characteristics, and treatment approaches likely contribute to the variability. Future research should consider stratified analyses by tumor type, stage, and treatment to identify sources of heterogeneity and provide more clinically meaningful survival estimates.

### Subgroup Analysis of Ovarian Cancer Survival Rates by Geographical Region and Period of Publication

#### Subgroup Analysis of 1-Year Ovarian Cancer Survival Rate by Geographical Region and Period of Publication

The 1-year ovarian cancer survival rate subgroup analysis based on African regions revealed notable disparities in ovarian cancer survival rates. Northern Africa demonstrated the highest pooled survival rate at 79.6% (95% CI 73.1–86.1%), followed closely by Eastern Africa with 76.8% (95% CI 71.6–82.0%), indicating better prognostic outcomes in these regions, likely reflecting relatively stronger cancer care infrastructure and earlier detection services. Southern Africa showed a moderately lower pooled survival rate of 62.8% (95% CI 47.4–78.2%), suggesting possible gaps in health system performance or access to advanced treatment. In contrast, Western Africa had the lowest pooled survival rate at 30.0% (95% CI 17.8–42.2%), underscoring critical challenges in cancer care delivery, early diagnosis, and healthcare resources. These regional differences emphasize the need for targeted, context-specific interventions to reduce disparities and improve survival outcomes across the continent (Supplementary S4 file Fig. 1).

The meta-analysis revealed significant variations in the pooled survival rate across different time periods. Studies conducted between 2020 and 2024 showed a pooled survival rate of 72.78 (95% CI 65.23–80.33%), while those from 2015 to 2019 yielded a slightly higher pooled survival rate of 78.50 (95% CI 73.23–83.77%). In contrast, the earliest subgroup (2010–2014) exhibited the lowest and most variable pooled survival rate at 56.50 (95% CI 31.62–81.38%). The trend suggests more consistent and higher pooled survival rates in recent years (2015–2024) compared with the earlier timeframe, which displayed wider confidence intervals and greater dispersion. These differences may reflect evolving methodologies, changes in population characteristics, or improvements in measurement techniques over time. A random-effects model was used to account for the high between-study variability (Supplementary S4 file Fig. 2).

#### Subgroup Analysis of 2-Year Ovarian Cancer Survival Rate by Geographic Region and Period of Publication

The meta-analysis demonstrated considerable variation in pooled survival rates across different African regions. Northern Africa exhibited the highest pooled estimate at 75.85% (95% CI 65.23–86.47%), with individual study estimates ranging from 39.60 to 97.00%. Eastern Africa showed moderate estimates with a pooled value of 68.83% (95% CI 59.15–78.51%), while Southern and Western Africa demonstrated lower pooled estimates of 43.70% (95% CI 27.93–59.47%) and 74.00% (95% CI 67.59–80.41%), respectively.

The random-effects model analysis revealed that Northern African studies contributed the greatest cumulative weight (34.27%), followed by Eastern Africa (49.85%). These regional disparities may reflect differences in healthcare infrastructure, cultural practices, or methodological approaches across studies. The overall pooled estimate across all regions was 69.08% (95% CI 57.31–80.85%), demonstrating moderate effectiveness with wide confidence intervals, likely due to the substantial heterogeneity observed (Supplementary S4 file Fig. 3).

The meta-analysis demonstrated notable variations in pooled survival rates when stratified by study period. The 2015–2019 subgroup showed the highest pooled survival rate (75.35%; 95% CI 58.42–92.28%), followed by the 2020–2024 subgroup (68.20%; 95% CI 55.14–81.26%), while the 2010–2014 subgroup yielded more conservative estimates (61.00%; 95% CI 48.73–73.27%). The random-effects model accounted for this variability, with studies from 2015 to 2019 contributing the largest weights to the overall analysis (Supplementary S4 file, Fig. 4).

#### Subgroup Analysis of 3-Year Ovarian Cancer Survival Rate by Geographical Region and Period of Publication

The meta-analysis results demonstrated substantial geographic variation in effect estimates across African regions. Northern African studies exhibited the most pronounced variability, with estimates ranging from 33.00% (95% CI 23.69–42.31%) to 97.00% (95% CI 92.88–100%). This region contributed nearly half of the total weight (49.96%) to the analysis, reflecting its strong representation in the included studies. Western African studies showed greater consistency, with estimates clustering between 39.50% and 63.60%, while the single Sub-Saharan African study reported an estimate of 54.40% (95% CI 50.60–58.20%) that closely approximated the overall pooled result. The findings highlight how regional disparities in healthcare systems, cultural contexts, and study methodologies can significantly influence outcomes, emphasizing the need for region-specific interpretations of results rather than relying solely on pooled estimates. These geographic variations may reflect differences in disease burden, intervention implementation, or healthcare access across the diverse African continent (Supplementary S4 file Fig. 5).

The meta-analysis results demonstrated significant variations in pooled survival rate between the two time periods examined. The average pooled survival rate for studies published between 2020 and 2024 was 61.49% (95% CI 45.19–77.80%) and the average pooled survival rate for studies published between 2015 and 2019 was 88.75% (95% CI 83.12–94.38%) (Supplementary S4 file Fig. 6).

#### Subgroup Analysis of 5-Year Ovarian Cancer Survival by Geographical Region and Period of Publication

This meta-analysis revealed substantial regional differences in effect estimates, with Northern Africa demonstrating the highest pooled effectiveness (72.4%; 95% CI 64.1–80.7%), followed by Western Africa (63.8%; 95% CI 52.4–75.2%) and Eastern Africa (55.1%; 95% CI 47.3–62.9%). Sub-Saharan Africa showed the most conservative estimate (45.0%; 95% CI 41.2–48.8%). Northern Africa’s superior performance may reflect more established health systems and greater resource availability, while Western Africa’s intermediate position shows notable country-level variations. Eastern Africa’s moderate estimates suggest relatively consistent but lower effectiveness than northern counterparts, and the limited Sub-Saharan data highlight a critical evidence gap (Supplementary S4 file Fig. 7).

The meta-analysis revealed significant temporal variations in pooled survival rate across the studied periods. The earliest period (2010–2014) demonstrated consistently high effectiveness, with a pooled survival rate of 85.00% (95% CI 76.45–93.55%). This period was characterized by uniformly strong results, potentially reflecting more selective study populations or different methodological approaches prevalent during these early years of research. The transitional period (2015–2019) showed remarkable variability, with pooled survival rate of 63.15% (95% CI 54.82–71.48%). This wide range suggests a period of methodological evolution, where changing study designs, varying implementation approaches, or shifts in population characteristics may have contributed to the observed dispersion in results. The most recent period (2020–2024) presented a more moderate pooled survival rate of 58.60% (95% CI 50.24–66.96%). While maintaining substantial variability, this period showed a general trend toward more conservative effectiveness estimates compared with earlier years (Supplementary S4 file Fig. 8).

#### Subgroup Analysis of 7-Year Ovarian Cancer Survival by Period of Publication

The pooled survival rate of 2020–2024 stood at 42.94% (95% CI 26.15–59.73%). The preceding 2015–2019 period showed improved outcomes with an average survival of 61.13% (95% CI 45.24–77.02%). Notably, the earliest available data, from 2010 to 2014, represented by a single study, reported a survival rate of 61.40% (95% CI 50.93–71.87%). The findings suggest that factors such as evolving methodologies, changes in healthcare delivery systems, or shifting population characteristics may contribute to these temporal differences. These temporal patterns underscore that healthcare interventions may not maintain consistent effectiveness over time, and pooled estimates alone may obscure important chronological trends that could inform policy and practice decisions (Supplementary S4 file Fig. 9).

### Publication Bias

Egger’s test is a statistical method used to detect publication bias in meta-analyses, with a *p*-value less than 0.05 indicating significant bias. A symmetric funnel plot, on the contrary, visually demonstrates that smaller studies are evenly distributed around the pooled effect size, indicating an absence of substantial publication bias. For the 1-year survival rate (*p* = 0.466), 3-year survival rate (*p* = 0.433), and 5-year survival rate (*p* = 0.477), the Egger’s test results are well above the 0.05 threshold, indicating no significant publication bias. This statistical result is reinforced by the observed symmetry of the funnel plots, confirming that smaller studies are not disproportionately skewed toward any particular effect size. For the 2-year (*p* = 0.063) and 7-year survival rates (*p* = 0.067), the *p* values are slightly above the significance cutoff, suggesting a possible but not statistically confirmed publication bias. However, the corresponding funnel plots remain symmetric, indicating that any potential bias is minimal and that the effect estimates are likely representative of the true survival rates. Overall, the combination of symmetric funnel plots and nonsignificant Egger’s test results across all timepoints strengthens the trustworthiness of our bias assessment. These findings suggest no strong evidence of publication bias, supporting the reliability of the pooled survival estimates reported in this meta-analysis (see Supplementary S5 file, Figs. 1, 2, 3, 4 and 5 for detailed visual results).

### Sensitivity Analysis

The sensitivity analyses for 1-year, 2-year, 3-year, 5-year, and 7-year survival rates among patients with ovarian cancer in Africa were conducted using a leave-one-out approach to assess the robustness of the pooled estimates. Although a visual plot was not included, the results of the leave-one-out sensitivity analyses are presented in detail in Supplementary Table S6 (Tables 1, 2, 3, 4 and 5). While the results generally show stability, some key variations were observed when specific studies were excluded, highlighting potential sources of heterogeneity.

For 1-year survival, the pooled estimate (71.41%, 95% CI 63.71–79.11%) remained largely unchanged when most studies were omitted. However, Iyoke et al. had the most notable impact, increasing the estimate to 76.86% (95% CI 73.15–80.57%), while Nabil et al. and Mlagalila et al. slightly reduced it to ~69.8%. This suggests that Iyoke et al. may have included a higher-survival cohort, but overall, the estimate is robust.

In the 2-year survival analysis, the pooled estimate (69.08%, 95% CI 57.31–80.85%) was most influenced by Elzarkaa et al. (71.80%, 95% CI 60.19–83.41%) and Lumley et al. (71.18%, 95% CI 59.10–83.26%), which pushed the survival rate higher. Conversely, Nassar et al. and Fayek et al. reduced the estimate to ~66.6%, indicating some variability in mid-term survival reporting.

The 3-year survival estimate (61.49%, 95% CI 45.19–77.80%) showed moderate sensitivity, with Sheta et al. (65.67%, 95% CI 49.20–82.13%) and Mazouzi et al. (64.23%, 95% CI 47.01–81.45%) increasing the estimate, while Gohar et al. (56.78%, 95% CI 47.14–66.43%) and Fayek et al. (58.77%, 95% CI 40.60–76.94%) lowered it. This suggests greater heterogeneity in longer-term survival data.

For 5-year survival, the pooled estimate (61.73%, 95% CI 52.72–70.74%) was relatively stable, but Abuidris et al. (63.18%, 95% CI 54.08–72.28%) and Mayenga et al. (62.77%, 95% CI 53.43–72.12%) slightly increased the survival rate, whereas Saber et al. (59.74%, 95% CI 51.31–68.18%) decreased it. The consistency in most exclusions supports the reliability of the 5-year estimate.

The 7-year survival analysis (53.74%, 95% CI 34.24–73.25%) revealed the highest variability, with Kamal et al. (61.67%, 95% CI 49.35–73.98%) substantially increasing the estimate, while Ali et al. (47.98%, 95% CI 27.78–68.17%) decreased it. This wide fluctuation suggests that long-term survival estimates are less reliable and may be influenced by differences in follow-up duration, treatment adherence, or regional disparities.

The exclusion of individual studies had minimal impact on the overall estimates for each survival period, indicating the consistency and reliability of the pooled survival rates across various timeframes. Minor fluctuations in survival estimates were observed in certain studies, likely due to differences in sample sizes, methodologies, or regional variations in treatment protocols. These findings underscore the robustness of the survival rate estimates and suggest that the results are reliable across different study populations and settings.

## Discussion

The results of this meta-analysis indicate that the estimated survival rates were 71.41% at 1 year, 69.08% at 2 years, 61.49% at 3 years, 61.73% at 5 years, and 53.74% at 7 years. The findings of this meta-analysis indicate that the estimated 1-year survival rate for ovarian cancer is 71.41%, which is consistent with previous study Asian countries.^72^ This finding is lower than that of previous studies conducted in the USA^73^ and in Brunei Darussalam.^74^ The justification might be earlier diagnosis and more comprehensive healthcare in the USA.^75^ In 2017, another study by Stewart et al. reviewed ovarian cancer survival rates between 2001 and 2009 in 37 states, which includes 80% of the population of the USA. The survival rate for ovarian cancer in women in the USA between 15 and 90 years old was 72.3% from 2001 to 2003 and increased to 73.3% from 2004 to 2009.^76^

The findings of this meta-analysis indicate that the estimated 2-year survival rate of ovarian cancer in Africa is 69.08%. This finding is consistent with earlier studies in Brunei Darussalam.^74^ The possible justification may be that they have comparable treatment modalities, health-seeking behaviors, and balancing socioeconomic status.

When compared with previous studies, survival rates in high-income regions tend to be higher. Studies conducted in the USA and Europe have reported 2-year survival rates exceeding 75–80%, largely due to earlier diagnosis through efficient diagnostic pathways, timely referral, and access to comprehensive multimodal treatment, including optimal cytoreductive surgery and chemotherapy.^77^ The United Kingdom Collaborative Trial of Ovarian Cancer screening (UKCTOCS) reported that early detection significantly enhances survival, with women diagnosed at early stages demonstrating 2-year survival rates above 80%.^78^ In Africa, late-stage diagnosis remains a major challenge, primarily due to low awareness of ovarian cancer symptoms, delayed health-seeking behavior, cultural perceptions and stigma surrounding cancer, financial and geographic barriers, and limited access to specialized gynecologic oncology services.^79^ Limited access to optimal treatment modalities, including cytoreductive surgery and platinum-based chemotherapy, significantly affects survival outcomes.^80,81^

At 3 years, the survival rate drops to 61.49%, indicating a progressive decline in survival over time, likely due to the emergence of chemotherapy-resistant disease. This trend aligns with global findings, where 3-year survival rates for ovarian cancer typically range between 55% and 65%.^72,74,82^ However, the highest and lowest 3-year survival rates in our study were found to be 84% in Türkiye^83^ and 46.72% in India,^84^ respectively. The possible variation in the availability and standardization of treatment protocols significantly impacts survival outcomes. Türkiye may have higher adherence to international guidelines for ovarian cancer management, including advanced chemotherapy regimens and surgical interventions. In contrast, variations in treatment quality and accessibility across different regions may lead to less favorable outcomes.^85^

Moreover, the 5-year survival rate in this meta-analysis is 61.73%, which is comparable to global estimates, where survival varies from 30 to 50% in developing countries and up to 70% in high-income settings.^1,72,74,86–88^

This meta-analysis is lower than the survival rates reported in previous studies,^73,89,90^ which may be attributed to several factors. A retrospective study conducted in China, which analyzed 63 pathological cases of ovarian cancer from 2000 to 2018, reported a 5-year survival rate of 69% in patients who underwent surgery for treatment. The study also found an overall 5-year survival rate of 80% for all patients.^91^ Previous studies, particularly those conducted in high-income countries, often report higher survival rates due to access to advanced medical treatments, including newer chemotherapy regimens and targeted therapies such as PARP inhibitors. These treatments have been shown to improve survival outcomes, especially for patients with certain genetic mutations.^92^ In addition, research indicates that a significant proportion of patients relapse within 2 years, and this recurrence significantly reduces long-term survival.^92^ In lower-income countries, limited access to timely and appropriate treatment can result in delayed diagnoses and treatments, which may contribute to advanced-stage diagnoses and poorer survival rates. High-income countries, on the contrary, typically have better access to early detection and novel therapies, which can result in better survival outcomes.^94^

By 7 years, the survival rate further declines to 53.74%, indicating the long-term challenges of disease recurrence and progression. This is in line with findings from GLOBOCAN 2020, which reported 7-year survival rates ranging from 50 to 60% in developed countries and significantly lower rates in resource-limited settings.^1^ It is lower than earlier studies.^95^ The possible explanation may be that African populations may be linked to higher rates of treatment discontinuation, disease recurrence, and lack of long-term follow-up programs.^96^ Additionally, the lack of advanced treatment options such as molecular-targeted therapy, maintenance therapy, and personalized cancer care strategies significantly contributes to poor long-term survival outcomes in Africa. Studies have shown that patients receiving PARP inhibitors as maintenance therapy experience prolonged progression-free survival, but these treatments remain inaccessible in most African settings due to cost and limited supply chains.^97^ In addition to differences in access to chemotherapy and diagnostic services, disparities in access to high-quality, specialized surgical care significantly influence ovarian cancer survival in Africa. Evidence from global oncology studies shows that many low- and middle-income countries face a shortage of trained gynecologic oncologists, limited opportunities to perform extensive cytoreductive surgery, and insufficient multidisciplinary care teams.^30,98^ The wide range of survival estimates across studies reflects substantial heterogeneity, likely due to differences in tumor type, stage at diagnosis, treatment modalities, and healthcare access. Our pooled survival estimates (71.41% at 1 year, 69.08% at 2 years, 61.49% at 3 years, 61.73% at 5 years, and 53.74% at 7 years) provide an overall picture but should be interpreted with caution, as they combine heterogeneous patient populations. This highlights the need for stratified analyses in future studies to identify subgroup-specific survival and guide targeted interventions.

Our subgroup analysis identified substantial regional disparities in ovarian cancer survival rates across Africa. Northern Africa consistently showed the highest survival rates (79.6% for 1-year survival), likely due to stronger healthcare infrastructure, better access to early diagnosis, and more advanced treatment options in this region. In contrast, Western Africa exhibited the lowest survival rates (30.0% for 1-year survival), reflecting critical challenges such as limited healthcare resources, delayed diagnosis, and insufficient cancer care services. These findings emphasize the significant influence of regional health system strength and resource availability on patient outcomes, underscoring the urgent need for region-specific interventions to address systemic inequities in cancer care. Temporal subgroup analyses revealed a decline in survival rates post-2019 compared with earlier periods, with 5-year survival dropping from 63.15% (2015–2019) to 58.60% (2020–2024). This decline may be explained by the impact of the coronavirus disease 2019 (COVID-19) pandemic, which disrupted healthcare services and delayed diagnosis and treatment across Africa. Additionally, changes in study methodologies and evolving patient demographics might have contributed to this trend. Notably, the 5-year survival rate was highest during 2010–2014 (85.0%), potentially reflecting small sample sizes, selective study populations, or methodological differences in earlier studies. In contrast, the 7-year survival rate declined markedly in the most recent period (2020–2024), with a pooled estimate of only 42.9%, possibly due to weakened health systems, delayed treatment, or disruptions such as the COVID-19 pandemic. Regionally, Northern Africa consistently showed superior 3- and 5-year survival outcomes, likely due to relatively stronger health infrastructure and better access to oncology services.

### Limitations

Although the included studies exhibited variability in study design and healthcare settings, the diversity in population characteristics is considered a strength of this review, as it enhances the generalizability of the findings across different contexts within Africa. However, the variation in study design, healthcare infrastructure, and the number and volume of studies conducted in each country may have influenced the overall survival estimates. Additionally, the absence of published studies on ovarian cancer survival from several African countries limits the completeness of the continental picture. Differences in diagnostic capabilities, treatment accessibility, and follow-up durations across regions may also contribute to heterogeneity in the reported survival rates, underscoring the need for more standardized and representative research across underreported regions. Furthermore, this review included English-language studies only, which may have led to the exclusion of relevant studies published in other languages. A key limitation of this study is that the pooled survival estimates combine data from studies with heterogeneous populations, including differences in tumor types, disease stages, and treatment modalities. Consequently, the common survival rates reported in this meta-analysis may not reflect survival for any specific patient subgroup and should be interpreted with caution. Future research should consider stratifying survival by tumor type, stage, and treatment and include multiple languages to improve the comprehensiveness and representativeness of findings.

## Conclusions

This meta-analysis provides a comprehensive estimation of ovarian cancer survival rates in Africa, revealing significant variations over time. The pooled survival rates of 71.41% at 1 year, 69.08% at 2 years, 61.49% at 3 years, 61.73% at 5 years, and 53.74% at 7 years highlight both progress and persistent challenges, including late-stage diagnosis, limited access to advanced treatments, shortages of specialized surgical care, and broader healthcare disparities across the continent. The observed regional variations emphasize the importance of context-specific strategies. Improving ovarian cancer outcomes in Africa requires not only expanding access to chemotherapy and optimizing treatment protocols, but also strengthening health promotion, public education, and training of healthcare providers. Policymakers should prioritize raising awareness of cancer symptoms, enhancing early diagnosis through effective referral systems, investing in workforce capacity for specialized surgery and oncology care, and ensuring equitable access to quality cancer services. Future research should continue to identify region-specific barriers to care and evaluate the impact of targeted interventions, supporting evidence-based policies to improve survival and reduce disparities across the continent.

## Supplementary Information

Below is the link to the electronic supplementary material.

Supplementary file1 (DOCX 31 kb)

Supplementary file2 (DOCX 14 kb)

Supplementary file3 (DOCX 31 kb)

Supplementary file4 (DOCX 66 kb)

Supplementary file5 (DOCX 27 kb)

Supplementary file6 (DOCX 20 kb)

## Data Availability

The datasets used and analyzed during the current study are available from the manuscript or supplementary file.
