# Supplementary material for: Survival Patterns of Patients with Ovarian Cancer in Africa: Systematic Review and Meta-analysis
Source: Ann Surg Oncol. 2026 Mar 18;33(7):6654–70. doi: 10.1245/s10434-026-19413-7 (PMC13242442; doi:10.1245/s10434-026-19413-7)
Supplement: Supplementary file 2 — Supplementary file2 (DOCX 14 kb) [file 10434_2026_19413_MOESM2_ESM.docx]

| **Database** | **Concept** | **Search Terms Used** | **Records Retrieved** |
| --- | --- | --- | --- |
| **Google Scholar** | Disease | "ovarian cancer" OR "ovarian carcinoma" OR "ovarian neoplasms" | 500 |
|  | Outcome | "survival" OR "survival rate" OR "overall survival" OR "survival analysis" | 150 |
|  | Region | Africa OR "Sub-Saharan Africa" | 57 |
|  | Final combined search | "ovarian cancer" AND "survival" AND "Africa" | 1,300 |
| **PubMed (MEDLINE)** | Disease | "Ovarian Neoplasms"[MeSH] OR "ovarian cancer"[tw] OR "ovarian carcinoma"[tw] | 36 |
|  | Outcome | "Survival"[MeSH] OR "survival rate"[tw] OR "overall survival"[tw] OR "survival analysis"[tw] | 125 |
|  | Region | "Africa"[MeSH] OR Africa[tw] OR "Sub-Saharan Africa"[tw] | 13 |
|  | Final combined search | ("Ovarian Neoplasms"[MeSH] OR "ovarian cancer"[tw] OR "ovarian carcinoma"[tw]) AND ("Survival"[MeSH] OR "survival rate"[tw] OR "overall survival"[tw] OR "survival analysis"[tw]) AND ("Africa"[MeSH] OR Africa[tw] OR "Sub-Saharan Africa"[tw]) | 342 |
| **Other databases** (EMBASE, Web of Science, Hinari) | Disease, outcome, region | Database-specific subject headings and free-text terms adapted from PubMed strategy | **104** |

Supplementary File S3: Search Strategy and Results of Survival Patterns of Patients With Ovarian Cancer In Africa: Systematic Review And Meta-Analysis
