# Supplementary material for: Survival Patterns of Patients with Ovarian Cancer in Africa: Systematic Review and Meta-analysis
Source: Ann Surg Oncol. 2026 Mar 18;33(7):6654–70. doi: 10.1245/s10434-026-19413-7 (PMC13242442; doi:10.1245/s10434-026-19413-7)
Supplement: Supplementary file 3 — Supplementary file3 (DOCX 31 kb) [file 10434_2026_19413_MOESM3_ESM.docx]

| Author | | **Selection** | | | | | **Comparability** | **Outcome** | | | | **Total** |  | | | |
| --- | --- | --- | --- | --- | --- | --- | --- | --- | --- | --- | --- | --- | --- | --- | --- | --- |
| Mazouzi C,et al. | | Representativeness s (1) | SamPle size(1) | Non respondents (1) | | Rrisk factor (2) | Comparable, based on the study design or analysis. confounding factors are controlled (1) | Assessment of the outcome (2) | | Stastical test(1) | |  |  | | | |
| Lumley C,et al. | | 1 | 1 | 0 | | 1 | 1 | 1 | | 1 | | 6 |  | | | |
| Elashry R,et al. | | 1 | 1 | 1 | | 1 | 1 | 1 | | 1 | | 7 |  | | | |
| Sallam YA,et al. | | 1 | 1 | 1 | | 2 | 1 | 1 | | 1 | | 8 |  | | | |
| Nabil H,et al. | | 1 | 0 | 1 | | 1 | 1 | 1 | | 1 | | 8 |  | | | |
| Zuhdy M,et al. | | 1 | 1 | 0 | | 2 | 1 | 1 | | 1 | | 9 |  | | | |
| Nassar HR,et al. | | 1 | 1 | 1 | | 1 | 1 | 1 | | 1 | | 7 |  | | | |
| Bassiouny D,et al. | | 1 | 1 | 1 | | 2 | 1 | 1 | | 1 | | 8 |  | | | |
| Fayek IS,et al. | | 1 | 1 | 1 | | 1 | 1 | 1 | | 1 | | 7 |  | | | |
| Ali A,et al. | | 1 | 1 | 1 | | 2 | 1 | 1 | | 1 | | 8 |  | | | |
| Elzarkaa AA,et al. | | 1 | 0 | 1 | | 1 | 1 | 1 | | 1 | | 8 |  | | | |
| Kamal IM,et al. | | 1 | 1 | 0 | | 2 | 1 | 1 | | 1 | | 9 |  | | | |
| Amin NH,et al. | | 1 | 1 | 1 | | 2 | 1 | 1 | | 1 | | 8 |  | | | |
| Gohar S,et al. | | 1 | 0 | 1 | | 1 | 1 | 1 | | 1 | | 8 |  | | | |
| AbdelrahmanM,et al. | | 1 | 1 | 0 | | 2 | 1 | 1 | | 1 | | 9 |  | | | |
| Saber MM,et al. | | 1 | 1 | 0 | | 1 | 1 | 1 | | 1 | | 6 |  | | | |
| Sheta H,et al. | | 1 | 1 | 1 | | 1 | 1 | 1 | | 1 | | 7 |  | | | |
| Mostafa MF,et al. | | 1 | 1 | 1 | | 2 | 1 | 1 | | 1 | | 8 |  | | | |
| Abdel Ghany AE,et al. | | 1 | 0 | 1 | | 1 | 1 | 1 | | 1 | | 8 |  | | | |
| Piszczan S,et al. | | 1 | 1 | 0 | | 2 | 1 | 1 | | 1 | | 9 |  | | | |
| Konya WP,et al. | | 1 | 1 | 1 | | 2 | 1 | 1 | | 1 | | 8 |  | | | |
| Mayenga DB,et al. | | 1 | 0 | 1 | | 1 | 1 | 1 | | 1 | | 8 |  | | | |
| Cheserem EJ,et al. | | 1 | 1 | 0 | | 2 | 1 | 1 | | 1 | | 9 |  | | | |
| Mburu AW,et al. | | 1 | 1 | 0 | | 2 | 1 | 1 | | 1 | | 9 |  | | | |
| Mworia KM,et al. | | 1 | 1 | 1 | | 2 | 1 | 1 | | 1 | | 8 |  | | | |
| Mokomba A,et al. | | 1 | 1 | 1 | | 2 | 1 | 1 | | 1 | | 8 |  | | | |
| Ayogu ME,et al. | | 1 | 1 | 1 | | 2 | 1 | 1 | | 1 | | 8 | 1 | | | |
| Okunade KS,et al. | | 1 | 0 | 1 | | 1 | 1 | 1 | | 1 | | 8 | 1 | | | |
| Okunade KS,et al. | | 1 | 1 | 0 | | 2 | 1 | 1 | | 1 | | 9 | 1 | | | |
| Okunade KS,et al. | | 1 | 1 | 1 |  | | 2 | 1 | | 1 | 8 | | | 1 | 1 |  |
| Okunade KS,et al. | | 1 | 1 | 1 | 1 | | 1 | 1 | | 1 | 7 | |  | | |  |
| Okunade KS,et al. | 1 | | 0 | 1 | 2 | | 1 | | 1 | 1 | 7 | |  | | |  |
| Iyoke CA,et al. | 1 | | 1 | 0 | 2 | | 1 | | 1 | 1 | 7 | |  | | |  |
| Abuidris DO,et al. | 1 | | 1 | 1 | 2 | | 0 | | 1 | 1 | 7 | |  | | |  |
| Mlagalila NF,et al. | 1 | | 1 | 1 | 2 | | 2 | | 1 | 1 | 9 | |  | | |  |
| Gizaw M,et al. | 1 | | 1 | 0 | 2 | | 1 | | 1 | 1 | 9 | |  | | |  |
| Hegazi R,et al. | 1 | | 1 | 1 | 2 | | 1 | | 1 | 1 | 8 | |  | | |  |
| Habteyes AT,et al. | 1 | | 1 | 1 | 2 | | 1 | | 1 | 1 | 8 | |  | | |  |

Cross-sectional Studies:

Very Good Studies: 9 points

Good Studies: 7-8 points

Satisfactory Studies: 5-6 points

Unsatisfactory Studies: 0 to 4 points
