# Supplementary material for: Survival Patterns of Patients with Ovarian Cancer in Africa: Systematic Review and Meta-analysis
Source: Ann Surg Oncol. 2026 Mar 18;33(7):6654–70. doi: 10.1245/s10434-026-19413-7 (PMC13242442; doi:10.1245/s10434-026-19413-7)
Supplement: Supplementary file 4 — Supplementary file4 (DOCX 66 kb) [file 10434_2026_19413_MOESM4_ESM.docx]

Supplementary S3 file Fig 1: show the subgroup analysis by geographical region of 1-year survival rate among ovary cancer patients in Africa.

supplementary S 4 file fig 2: show the subgroup analysis by period of publication of 1-year survival rate among ovary cancer patients in Africa

supplementary S 4 file fig 3: show the subgroup analysis by geographical region of 2-year survival rate among ovary cancer patients in Africa

supplementary S 4 file fig 4: show the subgroup analysis by period of publication of 2-year survival rate among ovary cancer patients in Africa.

supplementary S 4 file fig 5: show the subgroup analysis by geographical region of 3-year survival rate among ovary cancer patients in Africa.

supplementary S 4 file fig 6: show the subgroup analysis by period of publication of 3-year survival rate among ovary cancer patients in Africa.

supplementary S 4 file fig 7: show the subgroup analysis by geographical region of 5-year survival rate among ovary cancer patients in Africa.

supplementary S 4 file fig 8: show the subgroup analysis by period of publication of 5-year survival rate among ovary cancer patients in Africa.

supplementary S 4 file fig 9: show the subgroup analysis by period of publication of 7-year survival rate among ovary cancer patients in Africa.
