# Supplementary material for: Survival Patterns of Patients with Ovarian Cancer in Africa: Systematic Review and Meta-analysis
Source: Ann Surg Oncol. 2026 Mar 18;33(7):6654–70. doi: 10.1245/s10434-026-19413-7 (PMC13242442; doi:10.1245/s10434-026-19413-7)
Supplement: Supplementary file 5 — Supplementary file5 (DOCX 27 kb) [file 10434_2026_19413_MOESM5_ESM.docx]

supplementary S 4 file fig 1 : Funnel plot of publication bias of 1- year survival rate among ovariancancer patents in Africa

supplementary S 4 file fig 2: Funnel plot of publication bias of 2- year survival rate among ovarian cancer patents in Africa

supplementary S 4 file fig 3: Funnel plot of publication bias of 3- year survival rate among ovarian cancer patents in Africa

supplementary S 4 file fig 4: Funnel plot of publication bias of 5- year survival rate among ovarian cancer patents in Africa

supplementary S 4 file fig 5: Funnel plot of publication bias of 7- year survival rate among ovarian cancer patents in Africa
