# Supplementary material for: Survival Patterns of Patients with Ovarian Cancer in Africa: Systematic Review and Meta-analysis
Source: Ann Surg Oncol. 2026 Mar 18;33(7):6654–70. doi: 10.1245/s10434-026-19413-7 (PMC13242442; doi:10.1245/s10434-026-19413-7)
Supplement: Supplementary file 6 — Supplementary file6 (DOCX 20 kb) [file 10434_2026_19413_MOESM6_ESM.docx]

Supplementary S6 file Table 1: shows the sensitivity analysis of 1- year survival rate among ovarian cancer patents in Africa.

| **Study omitted** | **Estimate (%)** | **95% CI (%)** |
| --- | --- | --- |
| Mazouzi C, et al. | 71.41 | 63.71–79.11 |
| Lumley C, et al. | 72.30 | 64.17–80.44 |
| Elashry R, et al. | 71.41 | 63.71–79.11 |
| Sallam YA, et al. | 71.41 | 63.71–79.11 |
| Nabil H, et al. | 69.88 | 61.25–78.50 |
| Zuhdy M, et al. | 71.41 | 63.71–79.11 |
| Nassar HR, et al. | 71.41 | 63.71–79.11 |
| Bassiouny D, et al. | 71.41 | 63.71–79.11 |
| Fayek IS, et al. | 71.41 | 63.71–79.11 |
| Ali A, et al. | 71.41 | 63.71–79.11 |
| Elzarkaa AA, et al. | 71.41 | 63.71–79.11 |
| Kamal IM, et al. | 71.41 | 63.71–79.11 |
| Amin NH, et al. | 71.41 | 63.71–79.11 |
| Gohar S, et al. | 71.41 | 63.71–79.11 |
| Abdelrahman M, et al. | 70.94 | 62.59–79.28 |
| Saber MM, et al. | 71.41 | 63.71–79.11 |
| Sheta H, et al. | 71.41 | 63.71–79.11 |
| Mostafa MF, et al. | 71.41 | 63.71–79.11 |
| Abdel Ghany AE, et al. | 71.41 | 63.71–79.11 |
| Piszczan S, et al. | 70.39 | 60.09–80.68 |
| Konya WP, et al. | 71.41 | 63.71–79.11 |
| Mayenga DB, et al. | 71.41 | 63.71–79.11 |
| Cheserem EJ, et al. | 71.41 | 63.71–79.11 |
| Mburu AW, et al. | 71.41 | 63.71–79.11 |
| Mworia KM, et al. | 71.41 | 63.71–79.11 |
| Mokomba A, et al. | 71.41 | 63.71–79.11 |
| Ayogu ME, et al. | 71.41 | 63.71–79.11 |
| Okunade KS, et al. | 71.41 | 63.71–79.11 |
| Okunade KS, et al. | 71.41 | 63.71–79.11 |
| Okunade KS, et al. | 71.41 | 63.71–79.11 |
| Okunade KS, et al. | 71.41 | 63.71–79.11 |
| Okunade KS, et al. | 71.41 | 63.71–79.11 |
| Iyoke CA, et al. | 76.86 | 73.15–80.57 |
| Abuidris DO, et al. | 71.41 | 63.71–79.11 |
| Mlagalila NF, et al. | 69.71 | 61.13–78.29 |
| Gizaw M, et al. | 70.66 | 60.47–80.86 |
| Hegazi R, et al. | 69.56 | 61.05–78.07 |
| Habteyes AT, et al. | 71.41 | 63.71–79.11 |
| **Combined** | 71.41 | 63.71–79.11 |

Supplementary S6 file Table 2: shows the sensitivity analysis of 2- year survival rate among ovarian cancer patents in Africa.

| **Study omitted** | **Estimate (%)** | **95% CI (%)** |
| --- | --- | --- |
| Mazouzi C, et al. | 69.08 | 57.31–80.85 |
| Lumley C, et al. | 71.18 | 59.10–83.26 |
| Elashry R, et al. | 69.08 | 57.31–80.85 |
| Sallam YA, et al. | 69.08 | 57.31–80.85 |
| Nabil H, et al. | 69.08 | 57.31–80.85 |
| Zuhdy M, et al. | 69.08 | 57.31–80.85 |
| Nassar HR, et al. | 66.60 | 54.17–79.04 |
| Bassiouny D, et al. | 69.08 | 57.31–80.85 |
| Fayek IS, et al. | 66.41 | 54.10–78.72 |
| Ali A, et al. | 69.08 | 57.31–80.85 |
| Elzarkaa AA, et al. | 71.80 | 60.19–83.41 |
| Kamal IM, et al. | 69.08 | 57.31–80.85 |
| Amin NH, et al. | 69.08 | 57.31–80.85 |
| Gohar S, et al. | 69.08 | 57.31–80.85 |
| Abdelrahman M, et al. | 69.08 | 57.31–80.85 |
| Saber MM, et al. | 69.08 | 57.31–80.85 |
| Sheta H, et al. | 69.08 | 57.31–80.85 |
| Mostafa MF, et al. | 68.79 | 56.13–81.44 |
| Abdel Ghany AE, et al. | 69.08 | 57.31–80.85 |
| Piszczan S, et al. | 70.06 | 58.02–82.11 |
| Konya WP, et al. | 68.72 | 56.26–81.18 |
| Mayenga DB, et al. | 69.08 | 57.31–80.85 |
| Cheserem EJ, et al. | 70.93 | 59.27–82.58 |
| Mburu AW, et al. | 69.40 | 57.11–81.69 |
| Mworia KM, et al. | 67.04 | 54.48–79.61 |
| Mokomba A, et al. | 68.58 | 55.67–81.49 |
| Ayogu ME, et al. | 69.08 | 57.31–80.85 |
| Okunade KS, et al. | 69.08 | 57.31–80.85 |
| Okunade KS, et al. | 69.08 | 57.31–80.85 |
| Okunade KS, et al. | 69.43 | 56.99–81.87 |
| Okunade KS, et al. | 69.08 | 57.31–80.85 |
| Okunade KS, et al. | 69.08 | 57.31–80.85 |
| Iyoke CA, et al. | 69.08 | 57.31–80.85 |
| Abuidris DO, et al. | 69.08 | 57.31–80.85 |
| Mlagalila NF, et al. | 69.08 | 57.31–80.85 |
| Gizaw M, et al. | 69.08 | 57.31–80.85 |
| Hegazi R, et al. | 69.08 | 57.31–80.85 |
| Habteyes AT, et al. | 69.08 | 57.31–80.85 |
| **Combined** | 69.08 | 57.31–80.85 |

Supplementary S6 file Table 3: shows the sensitivity analysis of 3- year survival rate among ovarian cancer patents in Africa.

| **Study omitted** | **Estimate (%)** | **95% CI (%)** |
| --- | --- | --- |
| Mazouzi C, et al. | 64.23 | 47.01–81.45 |
| Lumley C, et al. | 61.49 | 45.19–77.80 |
| Elashry R, et al. | 61.49 | 45.19–77.80 |
| Sallam YA, et al. | 61.49 | 45.19–77.80 |
| Nabil H, et al. | 61.49 | 45.19–77.80 |
| Zuhdy M, et al. | 61.49 | 45.19–77.80 |
| Nassar HR, et al. | 61.49 | 45.19–77.80 |
| Bassiouny D, et al. | 61.49 | 45.19–77.80 |
| Fayek IS, et al. | 58.77 | 40.60–76.94 |
| Ali A, et al. | 61.49 | 45.19–77.80 |
| Elzarkaa AA, et al. | 61.49 | 45.19–77.80 |
| Kamal IM, et al. | 61.49 | 45.19–77.80 |
| Amin NH, et al. | 61.49 | 45.19–77.80 |
| Gohar S, et al. | 56.78 | 47.14–66.43 |
| Abdelrahman M, et al. | 61.49 | 45.19–77.80 |
| Saber MM, et al. | 61.49 | 45.19–77.80 |
| Sheta H, et al. | 65.67 | 49.20–82.13 |
| Mostafa MF, et al. | 61.49 | 45.19–77.80 |
| Abdel Ghany AE, et al. | 61.49 | 45.19–77.80 |
| Piszczan S, et al. | 61.49 | 45.19–77.80 |
| Konya WP, et al. | 61.49 | 45.19–77.80 |
| Mayenga DB, et al. | 61.49 | 45.19–77.80 |
| Cheserem EJ, et al. | 61.49 | 45.19–77.80 |
| Mburu AW, et al. | 61.49 | 45.19–77.80 |
| Mworia KM, et al. | 61.49 | 45.19–77.80 |
| Mokomba A, et al. | 61.49 | 45.19–77.80 |
| Ayogu ME, et al. | 61.49 | 45.19–77.80 |
| Okunade KS, et al. | 61.49 | 45.19–77.80 |
| Okunade KS, et al. | 61.73 | 43.38–80.07 |
| Okunade KS, et al. | 61.49 | 45.19–77.80 |
| Okunade KS, et al. | 61.10 | 42.27–79.93 |
| Okunade KS, et al. | 61.48 | 43.39–79.57 |
| Iyoke CA, et al. | 61.49 | 45.19–77.80 |
| Abuidris DO, et al. | 61.49 | 45.19–77.80 |
| Mlagalila NF, et al. | 61.49 | 45.19–77.80 |
| Gizaw M, et al. | 62.47 | 43.69–81.25 |
| Hegazi R, et al. | 61.49 | 45.19–77.80 |
| Habteyes AT, et al. | 61.49 | 45.19–77.80 |
| **Combined** | 61.49 | 45.19–77.80 |

Supplementary S6 file Table 4: shows the sensitivity analysis of 5- year survival rate among ovarian cancer patents in Africa.

| **Study omitted** | **Estimate (%)** | **95% CI (%)** |
| --- | --- | --- |
| Mazouzi C, et al. | 61.73 | 52.72–70.74 |
| Lumley C, et al. | 61.73 | 52.72–70.74 |
| Elashry R, et al. | 60.93 | 51.65–70.21 |
| Sallam YA, et al. | 60.24 | 51.97–68.51 |
| Nabil H, et al. | 61.73 | 52.72–70.74 |
| Zuhdy M, et al. | 60.79 | 51.53–70.06 |
| Nassar HR, et al. | 62.36 | 52.87–71.85 |
| Bassiouny D, et al. | 61.24 | 51.77–70.71 |
| Fayek IS, et al. | 62.40 | 53.05–71.76 |
| Ali A, et al. | 61.73 | 52.72–70.74 |
| Elzarkaa AA, et al. | 61.73 | 52.72–70.74 |
| Kamal IM, et al. | 61.73 | 52.72–70.74 |
| Amin NH, et al. | 61.73 | 52.72–70.74 |
| Gohar S, et al. | 61.73 | 52.72–70.74 |
| Abdelrahman M, et al. | 61.73 | 52.72–70.74 |
| Saber MM, et al. | 59.74 | 51.31–68.18 |
| Sheta H, et al. | 61.93 | 52.52–71.35 |
| Mostafa MF, et al. | 61.73 | 52.72–70.74 |
| Abdel Ghany AE, et al. | 61.73 | 52.72–70.74 |
| Piszczan S, et al. | 61.73 | 52.72–70.74 |
| Konya WP, et al. | 62.07 | 52.73–71.41 |
| Mayenga DB, et al. | 62.77 | 53.43–72.12 |
| Cheserem EJ, et al. | 61.73 | 52.72–70.74 |
| Mburu AW, et al. | 61.73 | 52.72–70.74 |
| Mworia KM, et al. | 60.64 | 51.40–69.88 |
| Mokomba A, et al. | 62.74 | 53.34–72.14 |
| Ayogu ME, et al. | 60.38 | 51.24–69.53 |
| Okunade KS, et al. | 61.61 | 52.27–70.94 |
| Okunade KS, et al. | 61.73 | 52.72–70.74 |
| Okunade KS, et al. | 61.73 | 52.72–70.74 |
| Okunade KS, et al. | 62.84 | 53.47–72.20 |
| Okunade KS, et al. | 61.73 | 52.72–70.74 |
| Iyoke CA, et al. | 61.73 | 52.72–70.74 |
| Abuidris DO, et al. | 63.18 | 54.08–72.28 |
| Mlagalila NF, et al. | 61.73 | 52.72–70.74 |
| Gizaw M, et al. | 62.77 | 53.23–72.31 |
| Hegazi R, et al. | 61.73 | 52.72–70.74 |
| Habteyes AT, et al. | 62.45 | 52.59–72.32 |
| **Combined** | 61.73 | 52.72–70.74 |

Supplementary S6 file Table 5: shows the sensitivity analysis of 7- year survival rate among ovarian cancer patents in Africa.

| **Study omitted** | **Estimate (%)** | **95% CI (%)** |
| --- | --- | --- |
| Mazouzi C, et al. | 53.74 | 34.24–73.25 |
| Lumley C, et al. | 53.74 | 34.24–73.25 |
| Elashry R, et al. | 53.74 | 34.24–73.25 |
| Sallam YA, et al. | 53.74 | 34.24–73.25 |
| Nabil H, et al. | 53.74 | 34.24–73.25 |
| Zuhdy M, et al. | 53.74 | 34.24–73.25 |
| Nassar HR, et al. | 53.74 | 34.24–73.25 |
| Bassiouny D, et al. | 53.74 | 34.24–73.25 |
| Fayek IS, et al. | 55.99 | 31.19–80.79 |
| Ali A, et al. | 47.98 | 27.78–68.17 |
| Elzarkaa AA, et al. | 53.74 | 34.24–73.25 |
| Kamal IM, et al. | 61.67 | 49.35–73.98 |
| Amin NH, et al. | 51.27 | 27.57–74.96 |
| Gohar S, et al. | 53.74 | 34.24–73.25 |
| Abdelrahman M, et al. | 53.74 | 34.24–73.25 |
| Saber MM, et al. | 53.74 | 34.24–73.25 |
| Sheta H, et al. | 53.74 | 34.24–73.25 |
| Mostafa MF, et al. | 53.74 | 34.24–73.25 |
| Abdel Ghany AE, et al. | 51.88 | 27.64–76.11 |
| Piszczan S, et al. | 53.74 | 34.24–73.25 |
| Konya WP, et al. | 53.74 | 34.24–73.25 |
| Mayenga DB, et al. | 53.74 | 34.24–73.25 |
| Cheserem EJ, et al. | 53.74 | 34.24–73.25 |
| Mburu AW, et al. | 53.74 | 34.24–73.25 |
| Mworia KM, et al. | 53.74 | 34.24–73.25 |
| Mokomba A, et al. | 53.74 | 34.24–73.25 |
| Ayogu ME, et al. | 53.74 | 34.24–73.25 |
| Okunade KS, et al. | 53.74 | 34.24–73.25 |
| Okunade KS, et al. | 53.74 | 34.24–73.25 |
| Okunade KS, et al. | 53.74 | 34.24–73.25 |
| Okunade KS, et al. | 53.74 | 34.24–73.25 |
| Okunade KS, et al. | 53.74 | 34.24–73.25 |
| Iyoke CA, et al. | 53.74 | 34.24–73.25 |
| Abuidris DO, et al. | 53.74 | 34.24–73.25 |
| Mlagalila NF, et al. | 53.74 | 34.24–73.25 |
| Gizaw M, et al. | 53.74 | 34.24–73.25 |
| Hegazi R, et al. | 53.74 | 34.24–73.25 |
| Habteyes AT, et al. | 53.74 | 34.24–73.25 |
| **Combined** | 53.74 | 34.24–73.25 |
